# Vertebral Osteomyelitis and Infective Endocarditis Co-Infection

**DOI:** 10.3390/jcm11082266

**Published:** 2022-04-18

**Authors:** Tadatsugu Morimoto, Hirohito Hirata, Koji Otani, Eiichiro Nakamura, Naohisa Miyakoshi, Yoshinori Terashima, Kanichiro Wada, Takaomi Kobayashi, Masatoshi Murayama, Masatsugu Tsukamoto, Masaaki Mawatari

**Affiliations:** 1 Department of Orthopedic Surgery, Faculty of Medicine, Saga University, 5-1-1 Nabeshima, Saga 849-8501, Japan; h.hirata.saga@gmail.com (H.H.); takaomi_920@yahoo.co.jp (T.K.); mountain_village_converse@yahoo.co.jp (M.M.); masa2goo99@yahoo.co.jp (M.T.); mawatam@cc.saga-u.ac.jp (M.M.); 2 Department of Orthopedic Surgery, Fukushima Medical University School of Medicine, Fukushima 960-1295, Japan; kojiotani1964@gmail.com; 3 Department of Orthopedic Surgery, Faculty of Medicine, School of Medicine, University of Occupational and Environmental Health, 1-1 Iseigaoka, Yahatanishi-ku Kitakyushu, Fukuoka 807-8566, Japan; e-nak@med.uoeh-u.ac.jp; 4 Department of Orthopedic Surgery, Faculty of Medicine, School of Medicine, Akita University, 1-1 Tegata Gakuen Town, Akita 010-8543, Japan; miyakosh@doc.med.akita-u.ac.jp; 5 Department of Orthopedic Surgery, School of Medicine, Sapporo Medical University, S1W17, Chuo-ku, Sapporo 060-8543, Japan; ytera@zf6.so-net.ne.jp; 6 Department of Orthopedic Surgery, Faculty of Medicine, School of Medicine, Hirosaki University, 5 Zaifu-cho, Hirosaki 036-8563, Japan; wadak39@hirosaki-u.ac.jp

**Keywords:** vertebral osteomyelitis, infective endocarditis, co-infection

## Abstract

Many cases of vertebral osteomyelitis (VO) and infective endocarditis (IE) co-infection have been reported, and it has been recognized that attention should be paid to the possibility of both diseases co-existing during diagnosis and treatment. However, the incidence, clinical status, and outcomes of IE in patients with VO remain unclear. For this study, the eligibility criteria for patient recruitment included all cases of VO at the five medical university hospitals. Patients with a history of spinal surgery were excluded from this study. Echocardiography was routinely performed for all patients with VO. IE was diagnosed according to the modified Duke criteria for definite endocarditis. We analyzed demographic data, underlying conditions, clinical features, laboratory data, echocardiography, radiologic images, treatments, and outcomes. VO was diagnosed in 59 patients and IE was diagnosed in seven patients (12%). There were no significant differences in the clinical features, microorganisms, or radiographic status between the VO-IE co-infection and VO-only groups. In this study, using routine echocardiography for VO, the IE prevalence was 12%. The lack of specific clinical features and laboratory findings may hamper the diagnosis of IE. Therefore, clinicians are always required to suspect IE in patients with VO.

## 1. Introduction

The incidence of vertebral osteomyelitis (VO) has been rising over the past several decades because of the extended life expectancy and an increase in chronic debilitating diseases and patients with immunosuppression [1,2]. VO is often associated with distant site infections, such as urinary tract infections, infective endocarditis (IE), intestinal infections, and skin infections from the hematogenous pathway. However, whether VO is the result or cause of such issues is often unclear [1,2]. In particular, IE is associated with severe morbidity and potentially lethal diseases (mortality rate: up to 30%) and is more common in the elderly than in younger individuals [2,3,4]. Therefore, early diagnosis and treatment are crucial in both IE and VO. Although IE was considered a rare disease, since the association between IE and spinal disease (SD) was first reported in 1965, the frequency of VO-IE co-infections seems to be increasing [5,6,7,8,9,10,11,12,13], with reported rates of 3–33% (Table 1) [1,2,6,7,8,9,10,11,12,13]. However, while many spine surgeons focus on the treatment of VO, coexisting IE may often be overlooked, as it shares many similar symptoms with VO, such as back pain, fever, and general fatigue [1,2]. Many case reports and case series of VO-IE co-infection have been published by cardiologists [6,10] and internal medicine and infectious disease departments [7,8,11,12,13], with few such reports by spine surgeons [2]. Furthermore, most study designs of VO-IE co-infection have been single-hospital-based retrospective studies not using routine echocardiography to detect IE or routine spinal radiographical studies to diagnose VO (Table 1) [1,2,6,7,8,9,10,11,12,13]. Thus, the clinical status of VO-IE co-infection (i.e., its incidence, clinical features, risk factors, and outcomes) remains unclear. The present study investigated the incidence and risk factors of co-infection with VO and IE among patients admitted to and treated by a spine surgeon from the Orthopedic departments at five university hospitals in Japan. In addition, we prospectively investigated the clinical features of patients with VO-IE co-infection and VO-only using routinely performed echocardiography to detect IE.

## 2. Materials and Methods

### 2.1. Study Designs and Settings

This was a multicenter, prospective, observational cohort study. The eligibility criteria for patient recruitment included all cases of VO at the five medical university hospitals between April 2017 and March 2018. In the five medical university hospitals, the Division of Infectious Disease/Prevention and Control usually advised on the diagnosis of infectious diseases, including IE, and suggested appropriate antibiotics and treatment durations. Patients with a history of spinal surgery were excluded from this study. All patients were followed up for between six months and one year.

### 2.2. Patients’ Characteristics

Patients diagnosed with VO were reviewed for demographic data (sex, age, nosocomial infection), underlying conditions, presumed source of infection, clinical features (i.e., back pain, low back pain, and fever > 38 °C), neurological deficits, laboratory data (elevated leukocyte counts, elevated C-reactive protein levels), echocardiography, and in-hospital mortality. Nosocomial infection was defined as an infection acquired later than 48 h after admission to our hospital because of VO or IE and manifesting new symptoms and signs.

The diagnosis of VO was established based on a compatible clinical picture (back pain and signs of systemic inflammation), confirmation by radiologic studies (X-ray, computed tomography [CT], magnetic resonance imaging [MRI]), and isolation of the microbiologic specimen from blood specimens or a percutaneous bone biopsy. Patients were neurologically evaluated using the Frankel classification as follows [14]: grade A, complete paralysis; grade B, sensory function only below the injury level; grade C, incomplete motor function below the injury level; grade D, fair to good motor function below the injury level; grade E, normal. According to the Frankel grading system, the neurological status was divided into major deficit (ABC) and minor or no deficit (DE). The mode of onset was evaluated using the Kulowski classification [15] (acute, subacute, and insidious). Plain radiographs were evaluated using the modified Griffiths classification [16]: stage 1, early stage with narrowing of the intervertebral interval disc spaces, worn-eaten destruction of the vertebral endplates, and irregularities of the limbus vertebrae; stage 2, progressive (destructive) stage with bone destruction and peripheral reactive formation of new bone; and stage 3, healing (osteosclerotic) stage with osteosclerosis, formation of osteophytes, and block vertebrae. The abscess was investigated using MRI and included spondylodiscitis and epidural or paravertebral abscesses. IE was diagnosed according to the modified Duke criteria for definite endocarditis [17]. An analysis was performed for all transesophageal echocardiography (TEE) procedures performed by a senior cardiologist as a routine diagnostic tool. We analyzed the demographic data, underlying medical illness, vertebral level involved, predisposing cardiac risk factors, valve involvement, bacteriologic diagnosis, radiologic images, treatments, and outcomes. Patients were followed up for at least six months with careful attention to detect relapses. This study was approved by the Institutional Review Board of the authors’ affiliated institutions, and written informed consent was obtained from each patient before study participation.

### 2.3. Statistical Analyses

Continuous variables are expressed as mean, standard deviation, or median and range. Categorical variables are expressed as the number of cases (percentage). The chi-square test and Fisher’s exact test were used for between-group comparisons of categorical variables, and the Mann-Whitney U-test and Student T test were used for continuous variables. The threshold for statistical significance was set at *p* < 0.05.

## 3. Results

Fifty-nine patients were treated for VO after excluding any patients with tuberculosis (*n* = 1), post-spinal surgical site infection (*n* = 2), refusal to participate (*n* = 1), and insufficient data (*n* = 1). Spontaneous VO was diagnosed in 59 patients during the study period and IE was detected in 7 patients (12%). Among the seven patients with VO-IE co-infection, five were found to have IE after VO diagnosis, with an average diagnostic delay of five days (range: 1–16 days). Two patients were diagnosed with IE prior to VO. Three patients were diagnosed with TEE after non-diagnostic transthoracic echocardiography (TTE).

### 3.1. Baseline Characteristics of the VO-IE Co-Infection and VO-Only Groups

There were no significant differences between the groups in terms of demographic data, underlying conditions, presumed sources of infection, clinical features, or laboratory data (Table 2).

With regard to echocardiography, TTE was performed in 100% of the patients in both groups, and the rate of TEE use was significantly higher in the VO-IE co-infection group than in the VO-only group (43% vs. 0%, *p* = 0.001). Among the patients with VO-IE co-infection, three patients were diagnosed with TEE after nondiagnostic TTE. The types of infected valves included left-sided native disease (seven patients, including one with bilateral disease: mitral and tricuspid valves) and device-related (no patients). Three patients required cardiac surgery (valvuloplasty in two patients and prosthetic replacement in one patient). The in-hospital mortality rate was significantly higher in the VO-IE group than in the VO-only group (14% vs. 0%, *p* = 0.006).

### 3.2. Microbiologic Data of the VO-IE Co-Infection and VO-Only Groups

The detailed microbiological data are shown in Table 3. No significant differences were observed in the pathogen identification rate between the VO-IE co-infection and VO-only groups (86% vs. 83%, *p* = 0.84). Regarding blood cultures, the performance rate (100% vs. 94%, *p* = 0.51) and rate of positive blood cultures (86% vs. 44%, *p* = 0.05) were higher in the VO-only group than in the VO-IE co-infection group. The rate of blood cultures in the two sets (100% vs. 81%, *p* = 0.20) did not differ significantly between the groups. In addition, no significant difference was noted between the VO-IE co-infection and VO-only groups in the rates of positive percutaneous needle biopsy (75% vs. 61%, *p* = 0.60) and local excision (100% vs. 85%, *p* = 0.75). Gram-positive pathogens accounted for the majority of the pathogens in the groups (57% vs. 63%, *p* = 0.59). Among the gram-positive pathogens, *Staphylococcus* species (methicillin-sensitive *Staphylococcus aureus*, methicillin-resistant *Staphylococcus aureus*, methicillin-resistant *Staphylococcus epidermidis*, *Staphylococcus epidermidis*, and methicillin-resistant *staphylococci*/methicillin-resistant coagulase-negative *staphylococci*) were the most common microorganisms in both groups (100% [4/4 patients] vs. 76% [25/33 patients], *p* = 0.27).

### 3.3. VO Characteristics in the VO-IE Co-Infection and VO-Only Groups

There were no significant differences in neurological severity (Frankel A, B, C) or onset type (Kulowski classification) between the two groups. In both groups, the lumbar region was the most frequently involved region, followed by the thoracic and cervical regions. The plain radiographic findings according to the modified Griffiths classification showed that the VO-IE co-infection was stage 1 in all cases. Furthermore, stage 1 was the most common, followed by stages 2 and 3 in the VO-only group. There was no significant difference in the vertebral destruction type between the two groups, nor was there a significant difference in the ratio of spondylodiscitis and epidural or paravertebral abscesses. Antimicrobial therapy alone was more frequent in the VO-IE co-infection group than in the VO-only group (57% vs. 17%, *p* = 0.02). Instrumentation surgery was performed in 29% of the patients co-infected with VO-IE and 50% of the patients with VO only (*p* = 0.30).

## 4. Discussion

The current study is the first prospective study to compare the clinical status of patients with VO-IE co-infection and VO-only treated by spine surgeons in the orthopedic department of several university hospitals. As spine surgeons are representative clinicians involved in diagnosing and treating VO, we emphasized the significance of the features of VO-IE co-infection in this study. The features of our patients with VO-IE co-infection were similar to those described in previously published reports (Table 1) [1,2,6,7,8,9,10,11,12,13], commonly including an aging population, male predominance, frequent heart surgery, and life-threatening conditions.

### 4.1. Prevalence of VO-IE Co-Infection

Retrospective studies have reported that the prevalence of IE in patients with VO ranges between 2–31% [1,2,6,7,8,9,10,11,12,13]. The wide range of VO-IE co-infection rates may be attributed to (1) differences in the performance rate of routine echocardiography in IE and/or spinal MRI in VO depending on clinicians’ expertise in each clinical department; (2) patient-related differences due to aging on a global scale, as there is a relationship between older age and higher frequency of both VO and IE occurrence [1,2,3,4]; and (3) marked advances in and/or the much wider availability of imaging modalities, such as echocardiography, CT, and MRI [1,3,4]. In this prospective study using routine TTE in all patients with VO diagnosed by spine surgeons with spinal MRI or CT, the prevalence of IE was 12%. Behmanesh et al. [2] reported a prevalence of VO-IE co-infection of 33% in their prospective study with the routine implementation of TEE. Although the prevalence of VO-IE co-infection varies among reports due to institutional biases and regional frequencies of VO and IE, IE appears to be commonly diagnosed in patients with VO. In addition, the rate of pyogenic arthritis in patients with IE varies from 5% to 31% in previous studies [18]. These results indicate that clinicians involved in musculoskeletal care should recognize the importance of suspected complications of IE in cases of VO and pyogenic arthritis, and vice versa. The frequency of heart surgery and mortality from IE has commonly been reported to be 25–60% and 15–30%, respectively [3,4]. Similarly, in patients with VO-IE, the frequency of heart surgery and mortality was 57% in our study and 29–67% in previous studies, and the mortality rates were 14% in our study and 29–67% in previous studies [1,2,6,7,8,9,10,11,12,13]. Akiyama [1] showed that IE was a significant factor associated with increased VO-related mortality in a large-scale Japanese data set (*n* = 6807) and suggested that VO should be considered a fatal systemic disorder rather than a localized spinal disorder. Recent studies have indicated that early surgical intervention in IE is associated with a lower rate of adverse events, frequency of heart surgery, and mortality, further emphasizing the need for early diagnosis or ruling out IE in patients with VO [19,20].

With regard to echocardiography for early diagnosis, the reported sensitivity for TTE ranges from 40% to 65% and that for TEE ranges from 90% to 100% [5]. Therefore, Behmanesh et al. [2] suggested routinely performing TEE for the detection of IE in patients with VO. However, TEE is costly and invasive (carries a risk of oropharyngeal, esophageal, or gastric trauma), and requires technical and medical resources [2,21]. Therefore, the cheap, easy-to-use, and non-invasive TTE should be used first to exclude endocarditis as a complication or cause of infection. If IE cannot be detected with TTE in suspicious cases (i.e., those with a persistent fever of unknown origin, murmur, and evidence of endocardial involvement, such as mitral valve regurgitation), TEE should be performed as needed. Surprisingly, Yamashita [3] reported a patient with IE who had negative results in eight consecutive blood cultures and two TEEs. Thus, IE was conclusively diagnosed using valvular surgery alone, demonstrating the difficulty of correctly diagnosing IE.

### 4.2. A Comparison of the Clinical Findings between the VO-IE Co-Infection and VO-Only Groups

Knowing the precise clinical status of VO-IE co-infection is extremely useful for making an early definitive and differential diagnosis. Clinical findings suggestive of IE in patients with VO have been reported to be related to back pain [10], neurologic complications [8], cardiac comorbidities (e.g., valvular disease and pacemakers) [2,9,11], renal failure [2], dialysis [2,9], glomerulonephritis (reported incidence from 0% to 46%) [3], sepsis [2,9], and C-reactive protein levels [11]. Although cardiac comorbidities are a risk factor for IE, the risk of cardiac comorbidities was not assessed in our study because none of our patients had any cardiac comorbidities. However, the present study showed that there were no significant differences in sex, age, nosocomial infection, underlying conditions, presumed source of the infection, back pain (including low back pain), fever over 38 °C, neurologic deficit, elevated leukocyte count, or elevated C-reactive protein level between the VO-IE co-infection and VO-only groups. Findings such as back pain, renal failure, and sepsis related to a compromised host may be common to both VO-IE co-infection and VO-only, as they were risk factors for both groups. Although cardiac murmur auscultation is an unfamiliar examination for spine surgeons, cardiac murmurs are an important finding that can suggest a diagnosis of IE. However, it has been reported that 12–38% of patients with IE do not have cardiac murmurs [3,22,23]. Therefore, a lack of specific clinical features and laboratory examination findings with VO-IE co-infection compared to VO-only may hamper the diagnosis of IE.

### 4.3. A Comparison of the Microbiological Findings between the VO-IE Co-Infection and VO-Only Groups

According to the modified Duke’s IE criteria, the two major criteria of consecutive positive blood cultures and the presence of vegetation, usually detected by echocardiography, are used worldwide to diagnose IE [3,4,17]. Therefore, blood cultures may be as important as echocardiography as an indicator of IE-related bacteremia. Interestingly, patients with IE tended to have higher rates of positive blood cultures (*p* = 0.05). As reported by Yamamoto et al. [24], multiple sets of blood cultures should be performed for the early diagnosis of IE in patients with VO, although costs can be an issue.

In the present study, there was a marked change in the incidence of *Staphylococcus* spp. as the most common causative organism. This has recently been emphasized by several researchers [3,25,26]. However, Mulleman et al. [27] concluded that patients with *Streptococcus* and *Enterococcus* infections were more likely to have a combined infection with endocarditis than those with *Staphylococcus*. With the increased frequency of VO and the increased detection of VO-IE co-infection, *Staphylococcus* species may have shifted from being the most common causative organism of VO to being the most common causative organism of VO-IE co-infection.

Although the positive blood culture rates indicating bacteremia were 86% in the VO-IE co-infection group and 44% in the VO-only group, the rate of instrumentation surgery for VO treatment was 29% in the VO-IE co-infection group and 50% in the VO-only group. As the use of instrumentation surgery for bacteremia increases the risk of surgical site infection, these findings need to be noted. In this study, instrumentation surgery was indicated in 29% of VO-IE co-infected patients and 50% of VO-only patients (*p* = 0.30), and VO-IE patients tended to avoid instrumentation surgery; however, the difference was not statistically significant. In cases with Vo-IE co-infection, the potential risk of surgical infection is increased, which may be an important factor in preventing instrumentation surgery.

In recent years, testing methods with high detection rates for causative organisms, such as 16S rRNA sequencing and matrix-assisted laser desorption/ionization time-of-flight mass spectrometry, have been introduced to detect causative organisms that can not be identified by conventional methods in the blood cultures of IE patients [28]. Therefore, a negative blood culture using conventional methods does not rule out bacteremia. This fact should be considered when using spinal instrumentation in cases of VO.

Several limitations of the present study warrant mention. First, it included a small number of patients with IE. Although TEE was routinely performed in all cases in this prospective study, TTE was performed in only three cases. This may lead to a false-negative diagnosis of IE, as the diagnostic rate of TTE is lower than that of TEE [5]. Second, whether VO causes IE or vice versa was not resolved in this analysis. Third, the mortality due to VO-IE co-infection and VO-only may be underestimated because patients may have been transferred to other hospitals. Finally, there was institutional bias, as patients in university hospitals have a high number of comorbidities. Therefore, the representative value of this population may be limited. Despite these limitations, our study is the first prospective study to investigate the clinical status of patients with VO-IE co-infection treated by spine surgeons in the orthopedic department, and has provided several new findings regarding the incidence and clinical status of VO-IE co-infection.

## 5. Conclusions

In this prospective study in which TTE was routinely performed in all patients with VO diagnosed by spine surgeons via spinal MRI or CT, the prevalence of IE was 12%. Similar to previous reports on different subjects, IE may be commonly diagnosed with VO. The features of our patients with VO-IE co-infection included an aging population, male predominance, a high rate of positive blood cultures, frequent heart surgery, and life-threatening conditions.

The lack of specific clinical features and laboratory examination findings may make it more challenging to consider IE in patients with VO. Therefore, clinicians, especially spine surgeons and internal medicine physicians involved in VO practice, need to have a high awareness of IE to avoid missing this important complication and should routinely perform echocardiograms and multiple sets of blood cultures, as prompt diagnosis and supportive therapy are essential for minimizing morbidity and mortality. Further research in a larger patient population will help to clarify the clinical status of VO-IE co-infection.

## Figures and Tables

**Table 1:** Previous reports of vertebral osteomyelitis and infective endocarditis co-infection.

Author/Year	Department	Study Design	VO * Cases *n*	VO-IE * Cases % (*n*)	Male % (*n*)	Age Years (Range)	IE Surgery % (*n*)	Mortality Rate % (*n*)
Ninet [6] (1984)	Cardiology	Retrospective study	150	9 (14)	86 (12)	56.6 (39–72)	29 (4/14)	7 (1/14)
Morelli [7] (2001)	Clinica Medica	Retrospective study	30	10 (3)	100 (3)	48 (33–68)	67 (2/3)	0 (0/3)
Le Moal [8] (2002)	Internal Medicine and Infectious Diseases Department	Retrospective study	92	15 (14)	57 (8)	69.1 ± 13.6 (33–87)	57 (8/14)	7 (1/14)
Pigrau [9] (2005)	Infectious Disease Department	Retrospective study	91	31 (28)	75 (21)	66 ± 10.5	39 (11/28)	7 (2/28)
Tamura [10] (2010)	Cardiovascular Surgery	Retrospective study	77	13 (11)	72 (8)	61.2 ± 11.7 (42–81)	55 (6/11)	0 (0/11)
Akiyama [1] (2013)	Orthopedic Surgery	Retrospective study	6807	2 (145)	N/A	N/A	N/A	12 (18/145)
Koslow [11] (2014)	Internal Medicine	Retrospective study	62	23 (17)	77 (13)	70.6 ± 12.4	35 (6/17)	41 (7/17)
Murillo [12] (2018)	Infectious Diseases	Retrospective study	202	21 (43)	N/A	N/A	N/A	N/A
Behmanesh [2] (2019)	Neurosurgery	Prospective study	110	33 (36)	75 (27)	70.3 ± 7.4	33 (13/36)	22 (8/36)
Courjon [13] (2019)	Infectious Disease Department	Retrospective study	351	15 (51)	69 (242)	N/A	N/A	N/A

* VO, vertebral osteomyelitis; IE, infective endocarditis; N/A, not applicable.

**Table 2:** Baseline characteristics of the VO-IE co-infection and VO-only groups.

	VO-IE * Co-Infection	VO *-Only	*p*-Value
	N = 7 (12%)	N = 52 (88%)	
Demographic data			
male	5 (71%)	39 (75%)	0.84
Age (median in years)	76 ± 10.5	69 ± 12.4	0.32
nosocomial infection	1 (14%)	8 (15%)	0.94
Underlying conditions			
Diabetes mellitus	1 (14%)	15 (29%)	0.91
Neoplasm	3 (43%)	10 (19%)	0.16
Immunosuppression	1 (14%)	6 (12%)	0.83
Dialysis	0	2 (4%)	1.00
Bad teeth hygiene	1 (14%)	4 (8%)	0.56
Cardiac comorbidities	0	0	(-)
Presumed source of the infection			
Unknown	4 (42%)	41 (79%)	0.34
Skin and soft tissue	1 (14%)	1 (2%)	0.09
Pneumonia	1 (14%)	1 (2%)	0.09
Urinary tract	1 (14%)	5 (10%)	0.7
Digestive system	0	1 (2%)	1.00
Odontogenic	0	2 (4%)	1.00
Prosthetic knee	0	1 (2%)	1.00
Clinical features			
Back pain	4 (57%)	42 (81%)	0.16
Low back pain	4 (57%)	41 (79%)	0.21
Fever (>38 °C)	4 (57%)	23 (44%)	0.52
Laboratory data			
Leukocytosis (K/µL)	17.8 ± 4.2	11.9 ± 5.1	0.31
Elevated C-reactive protein (mg/dL)	16.2 ± 8.6	14.5 ± 7.5	0.18
Echocardiography			
Transthoracic echocardiography	7 (100%)	52 (100%)	1.00
Transesophageal echocardiography	3 (43%)	0 (0%)	0.001
Infected valve			
Mitral valve	1 (14%)	(-)	(-)
Aortic valve	3 (43%)	(-)	(-)
Mitral and aortic valve	1 (14%)	(-)	(-)
Mitral and tricuspid valve	1 (14%)	(-)	(-)
IE Treatment			
Conservative	4 (57%)	(-)	(-)
Cardiac surgery	3 (43%)	(-)	(-)
Valvuloplasty	2		
Prosthetic replacement	1		
In-hospital mortality	1 (14%)	0 (0%)	0.006

* VO, vertebral osteomyelitis; IE, infective endocarditis.

**Table 3:** Microbiological data of the VO-IE co-infection and VO-only groups.

	VO-IE * Co-Infection	VO *-Only	*p*-Value
	N = 7 (12%)	N = 52 (88%)	
**Pathogen identification, *n* (%)**	6 (86%)	43 (83%)	0.84
**Performance of blood cultures, *n* (%)**	7 (100%)	49 (94%)	0.51
positive blood cultures *n* (%)	6 (86%)	23 (44%)	0.05
blood cultures (over two sets)	7 (100%)	42 (81%)	0.2
**Performance of percutaneous needle biopsy and local excision at VO site, *n* (%)**	7 (100%)	43 (83%)	0.23
positive percutaneous needle biopsy and local excision at VO site, *n* (%)	6 (86%)	31 (72%)	0.18
positive percutaneous needle biopsy, *n* (%)	3/4 (75%)	14/23 (61%)	0.6
positive local excision, *n* (%)	3/3 (100%)	17/20 (85%)	0.47
**Microorganism**			
Gram-positive	4 (57%)	33 (63%)	0.75
MSSA *	2	12	0.75
MRSA *	1	6	0.83
MRSE *	0	2	0.6
*Staphylococcus epidermidis*	1	1	0.83
MRS/MSCNS *	0	4	0.45
*Streptococcus* species	0	5	0.39
*Aerococcus urinae*	0	1	0.71
*Candida albicans*	0	2	0.6
Gram-negative	2	10	0.56
*Enterococcus* spp.	2	4	0.09
*Klebsiella pneumonia*	0	3	0.51
*Salmonella*	0	1	0.71
*Pavinionas mircros*	0	1	0.71
*Proteus valgaris*	0	1	0.71

* VO, vertebral osteomyelitis; IE, infective endocarditis; MSSA, Methicillin-susceptible *Staphylococcus aureus*; MRSA, Methicillin-resistant *Staphylococcus aureus*; MRSE, Methicillin-resistant *Staphylococcus epidermidis*; MRS/MSCNS, Methicillin-resistant *Staphylococcus*/Methicillin-susceptible coagulase-negative *Staphilococci*.

## Data Availability

The data that support the findings of this study are available from our study Group, but restrictions apply to the availability of these data, and so are not publicly available. Data are, however, available from the authors upon reasonable request and with permission of our study Group.
